# Endosperm–Embryo Communications: Recent Advances and Perspectives

**DOI:** 10.3390/plants10112511

**Published:** 2021-11-19

**Authors:** Jingpu Song, Xin Xie, Yuhai Cui, Jitao Zou

**Affiliations:** 1 Aquatic and Crop Resource Development Research Centre, National Research Council of Canada, Saskatoon, SK S7N 0W9, Canada; Jitao.Zou@nrc-cnrc-gc.ca; 2 London Research and Development Centre, Agriculture and Agri-Food Canada, London, ON N5V 4T3, Canada; xxie226@uwo.ca (X.X.); yuhai.cui@canada.ca (Y.C.); 3 Department of Biology, Western University, London, ON N6A 3K7, Canada

**Keywords:** seed development, endosperm–embryo communications, mobile signals

## Abstract

Seed maturation depends on well-coordinated communications between the processes of endosperm and embryo development. The endosperm is considered to be destined to support embryo development and the timing of endosperm cellularization is critical for embryo growth. Recent findings suggest that the endosperm development and the onset of embryo maturation are two independent processes during seed development. Meanwhile, it is lately reported that several mobile regulators originating from the endosperm are needed to ensure proper embryo growth and seed maturation. In this opinion article, we highlight processes on how endosperm communicates with embryo during seed development and discuss some intriguing questions in light of the latest advancements.

## 1. Endosperm and Embryo, a Tale of Two Developments

Seed development begins with the formation of endosperm and embryo from the fertilized central cell and the fertilized egg cell (zygote), respectively. Although possessing the same genetic information, the triploid endosperm and the diploid embryo develop towards distinct destinations. In *Arabidopsis thaliana*, endosperm development undergoes syncytium formation, free nuclei cellularization, and degeneration stages [1,2,3]. In parallel, the zygote gives rise to the mature embryo through morphogenesis and maturation processes [4,5]. Although endosperm development and embryogenesis are two separate processes, proper seed development requires elaborated communications between the two. In this opinion review, we discuss whether endosperm development and embryogenesis require and affect each other based on several recent studies. We focus on several endosperm-originated protein signals, particularly transcription factors that play essential roles in endosperm–embryo communications during seed development. What we propose here could inspire some potential approaches to manipulate seed development, for instance, seed maturation and seed size, through modulating endosperm development process.

## 2. Initiation of Endosperm Development, Dependent or Independent on Egg Cell Fertilization?

**Figure 1:**
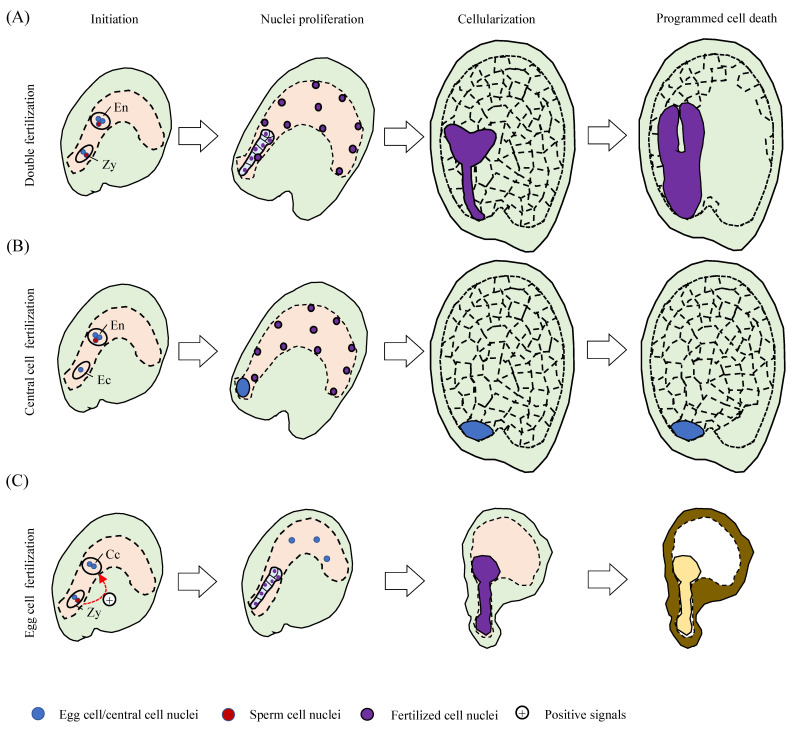
Models depicting the endosperm developments in double and single fertilization events. (**A**) In the double fertilization event (wild type), endosperm develops immediately after fertilization, undergoes nuclei proliferation, cellularization, followed by the programmed cell death (PCD) process. (**B**) In the single fertilization of a central cell (*dmp8/9*, or *gex2*) event, the triploid endosperm develops normally in nuclei proliferation and cellularization but shows a delayed PCD process. (**C**) In the single fertilization of an egg cell event, the unfertilized central cell is promoted for a slow nuclei proliferation process by a positive signal released from the zygote (fertilized egg cell). The fertilized embryo is finally arrested at the globular stage. En, endosperm; Zy, zygote; Ec, egg cell; Cc, central cell.

The onset of endosperm and embryo development follows the event of double fertilization (Figure 1A). Subsequent to the fertilization of the binucleate central cell with a sperm nucleus, the primary endosperm nucleus rapidly undergoes mitoses without cell division, leading to the syncytial endosperm stage including three domains that are termed as micropylar, chalazal and peripheral endosperm [6]. The micropylar endosperm nuclei surrounds the embryo and suspensor; the chalazal endosperm nuclei are at the chalazal pole opposite to the embryo; and the peripheral endosperm nuclei are spread as a peripheral layer. The micropylar and peripheral endosperm nuclei subsequently undergoes cellularization after eight rounds of mitoses, which in Arabidopsis takes place 4 days after pollination (DAP) [7,8]. Soon after cellularization at 5 DAP, the endosperm enters a phase of programmed cell death (PCD) that completes by 9 DAP [1]. During seed development, embryo development relies on endosperm development [3]. A corollary question of whether endosperm development requires developing embryo has also received considerable attention. 

Recent studies using single-fertilization mutants highlighted the independence and autonomous nature of endosperm development from the events of embryogenesis [1]. In Arabidopsis, lack of *GAMETE-EXPRESSED 2* (*GEX2*) or *DOMAIN OF UNKNOWN FUNCTION 679 membrane protein 8/9* (*DMP8/9*) causes singular fertilization with the central cell and leaves the egg cell unfertilized [9,10,11] (Figure 1B). In these mutants, endosperm initiation, syncytium formation, cellularization and degeneration could proceed in the absence of a zygote (embryo) during seed development [1]. Compared to the double-fertilization event, the single fertilization with the central cell only shows slow endosperm breakdown at the final developmental stage (Figure 1A,B). However, the real implication of these observations is that the initiation of normal endosperm development does not necessarily require egg cell fertilization. The single-fertilization event was also observed in the *cdka;1* mutant plants [12]. Lack of CDKA;1, one A-type cyclin-dependent kinase (CDK) in the pollen produces only one viable sperm cell which fertilizes the egg cell exclusively, giving rise to a developing embryo and unfertilized central cell in the embryo sac [12] (Figure 1C). Interestingly, the single-fertilization event that occurrs in the *cdka;1* mutant is different from that of the *gex2* or the *dmp8/9* mutant plants; the former preferably fertilizes egg cell and the latter only fertilizes the central cell. Both types of mutant exhibit fertility defects in the male, not the female [1,12], and it is not clear why they tend to have a preference of which cell to fertilize, the egg cell or the central cell. Although the central cell is not fertilized in the *cdka;1* plants, endosperm development does initiate after egg cell fertilization. Single fertilization of the egg cell by crossing *fis* mutant with *cdka;1* pollen promotes immediate endosperm development. Of note, Lack of *FERTILIZATION INDEPENDENT SEED* (*FIS*) genes in the *fis* family mutants’ endosperm develops autonomously but is retarded in unpollinated flowers [13]. It was thus proposed that the fertilization of the egg cell releases a positive signal to initiate proliferation of the unfertilized central cell [12]. In addition, development of *cdka;1* self-crossed seeds arrests at the globular stage, but crossing *fis* mutant with *cdka;1* pollens can produce small but viable seeds [14,15].

Hence, upon central cell fertilization, endosperm undergoes an autonomously programmed development process with or without a successful egg cell fertilization (Figure 1A,B). In the scenario where only the egg cell is fertilized, the zygote, in seeking support from endosperm tissues, sends out a signal to promote the initiation of central cell proliferation (Figure 1C). The initiation of endosperm development could be triggered by the central cell fertilization event or by a signal from the fertilized egg cell. Current data, therefore, suggest that the fertilized central cell development is a self-determining process independent of embryogenesis, but endosperm development from unfertilized central cell depends on the embryo development.

## 3. Endosperm and Embryo Communications: Much More Than Just Nutrients

That endosperm supports embryo growth and germination by providing nutrients and growth regulators has been intensively studied [2,16,17], and details of nutrient trafficking between endosperm and embryo has been previously reviewed [3]. With the discovery of small-interfering RNAs (SiRNA) travelling from the central cell to the egg cell, the prospect of epigenetic influence in the germ cell became apparent [18,19], and clearly endosperm offers embryo development more than just nutrients. In the following, we briefly summarize the recently discovered mobile protein regulators that are key to various seed developmental processes.

### 3.1. Endosperm-Synthesized LEC1, and Why It Matters

In terms of the time course of seed development process, endosperm cellularization occurs when an embryo enters the transition stage from morphogenesis to maturation. Published evidence indicate that failure of endosperm cellularization causes arrest of embryo development [13,20,21,22]. Interestingly, it was demonstrated that embryo arrest resulting from failure of endosperm cellularization could be bypassed by in vitro cultivation of dissected embryos [21]. Analysis of seed maturation markers in the Arabidopsis seeds shows that the onset of embryo maturation is mainly determined by the developmental stage of the embryo and does not require endosperm cellularization [23]. However, accumulating evidence has demonstrated that embryogenesis requires certain protein regulators originating from the endosperm [8,15,24].

**Figure 2:**
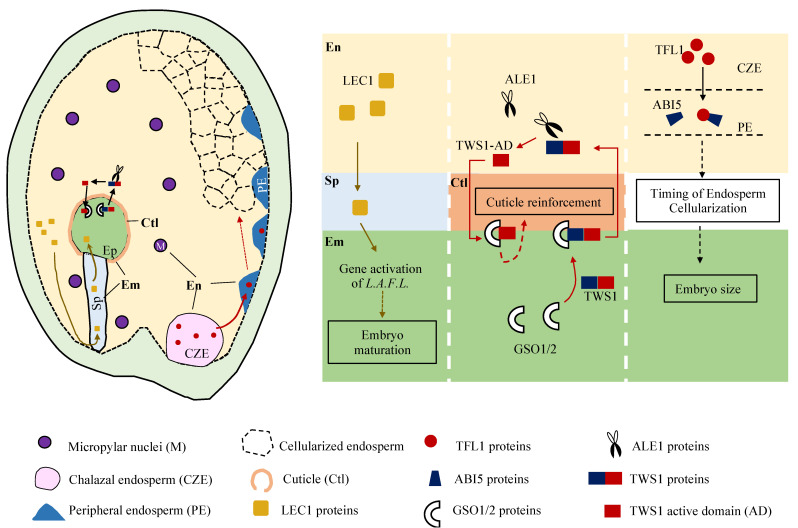
A schematic diagram showing the endosperm-originated regulators in determining embryo development. LEC1 is proposed to be initially expressed in the endosperm, then mobilized to the embryo proper through suspensor to trigger the gene activation of master regulators L. A. F. L (LEC1, ABI3, FUS3 and LEC2) involved in the embryo maturation process. TFL1 is expressed in the chalazal endosperm, then mobilized to the peripheral endosperm to stabilize ABI5 proteins, thus regulating the timing of endosperm cellularization which subsequently determines embryo size. TWS1 functions as a ligand of the receptor-like kinases GSSHO1/2 in the embryo. The sulfated peptide of TWS1 needs to be cleaved by ALE1 expressed in the endosperm cells to release the active peptide back to the embryo for cuticle reinforcement. Of note, these processes might occur at various seed development stages. En, endosperm; Em, embryo; Ep, embryo proper; Sp, suspensor.

LEAFY COTYLEDON1 (LEC1) is an essential transcription factor for seed maturation [25,26,27]. Lack of *LEC1* causes the failure of embryo maturation [15,25,26]. Our recent discovery shows that LEC1 expression can be detected as early as in the fertilized central cell nucleus prior to the embryo nuclei [15]. Furthermore, the absence of *LEC1* expression in the endosperm causes defective embryo development even in the presence of functional *LEC1* alleles in the embryo. Inversely, endosperm-synthesized LEC1 is fully capable of orchestrating seed maturation in the absence of embryo-expressed LEC1. Exclusive expression of *LEC1* in the endosperm restores the defected phenotypes of loss-of-function mutant *lec1* seeds [15]. This indicates that endosperm mobilizes the key embryo maturation regulator LEC1 to the embryo at a time point much earlier than endosperm cellularization. This could mean that, although embryo maturation is independent from endosperm cellularization, it does require the import of LEC1 from the endosperm to trigger the maturation process (Figure 2).

### 3.2. Timing the Endosperm Cellularization for Seed Sizes, When and How

Endosperm development impacts the final seed size by spatially confining the embryo growth as the result of endosperm expansion and integument elongation before endosperm cellularization [28]. The molecular mechanism controlling the timing of endosperm cellularization has become much clearer with the recent study of *TERMINAL FLOWER1* (*TFL1*) [8]. TFL1 is a phosphatidylethanolamine binding protein (PEBP) and is identified as an endosperm mobile signal. Loss-of-function mutant tfl1 delays endosperm cellularization, leading to a larger seed [8]. Further, available evidence showed that TFL1 is expressed in the chalazal endosperm, followed by trafficking to the syncytial peripheral endosperm which is mediated by a group of small GTP-binding Ras-related nuclear (RAN) proteins. TFL1 stabilizes ABI5 in the syncytial peripheral endosperm. ABI5 directly represses the expression of *SHORT HYPOCOTYL UNDER BLUE1* (*SHB1*), subsequently regulating the timing of endosperm cellularization [8,29]. In this scenario, carefully orchestrated timing of endosperm cellularization via protein trafficking of TFL1 in the endosperm likely serves as an important mechanism by which fine tuning of embryo growth and seed size is achieved (Figure 2). 

### 3.3. Working Together to Build Extra Cuticular Sheath, Where and Why

When endosperm cellularization occurs, an extracuticular sheath is deposited outside of the embryo cuticle, which is required for embryo–endosperm separation to ensure normal seed development. Sheath production depends upon the activity of an endosperm-specific bHLH transcription factor ZHOUPI (ZOU) [30,31], also known as RETARDED GROWTH OF EMBRYO1 (RGE1) [32]. Current evidence available show that ZOU controls specific signaling pathways that coordinate embryo expansion and endosperm breakdown, as well as triggering cell death by regulating the expression of cell-wall-modifying enzymes [30,33]. A recent study described a two-way communication between the endosperm and embryo, in which TWISTED SEED1 (TWS1) functions as a ligand of the receptor-like kinases GSSHO1 and GSSHO2 in the embryo and the sulfated peptide of TWS1 needs to be cleaved by the protease of the subtilase family ALE1 in the endosperm cells to release the active peptide back to the embryo, which then triggers GSSHO1/2-dependent cuticle reinforcement in the embryo [24]. Given the fact that the key enzymes TWS1 and ALE1 are separated by the cuticular sheath, this cuticle reinforcement action entails the teamwork of endosperm and embryo (Figure 2).

## 4. Underlying the Communications, Route and Means

In plants, neighboring cells can communicate via the apoplast or the symplast. Specific membrane transporters are required for the apoplastic transport, while plasmodesmata (PD) are involved in the symplastic transport pathway. The latter transportation supports both small molecules and macromolecules, including proteins or RNAs [34]. It is speculated that the communication between endosperm and embryo is mainly apoplastic during embryo development, while the transportation of signals between the suspensor and the embryo proper cells is mainly symplastic through PD in the early phase [3,35,36]. Our experimental evidence showed that the transcription factor LEC1 can be transported from endosperm to embryo (Figure 2). When LEC1 fused with one GFP is expressed exclusively in the endosperm, the GFP signals are also present in the suspensor and embryo. In contrast, signals of endosperm-expressed LEC1 fused with three GFPs are not seen in the suspensor and embryo [15]. These findings are consistent with the notion that the endosperm-originated transcription factor LEC1 enters suspensor and embryo through PD. TFL1 is another endosperm-originated mobile protein signal that is involved in determining embryo growth (Figure 2). Although TFL1 is not seen to transport from endosperm to embryo, the trafficking of TFL1 protein was observed from the chalazal endosperm where it is expressed to the syncytial peripheral endosperm where it is accumulated [8]. Further evidence suggested that TFL1 requires the nucleocytoplasmic transporter RAN1 for protein trafficking. 

The type of nutrient transport between endosperm and embryo changes as the seed develops. Before endosperm cellularization, the early embryo uptakes nutrients from the surrounding endosperm mostly through suspensor [37]. After endosperm cellularization, the embryo uptakes the nutrients directly from the endosperm as the suspensor degenerates [38]. This corresponds well with the reinforcement of the cuticle between the endosperm and the embryo. Interestingly, the reinforcement of the cuticle requires a bidirectional signal exchange between the two tissues (Figure 2). It is postulated that the sulfated TWS1 precursor is produced by the embryo and diffused to the endosperm via an apoplastic pathway with the absence of an intact cuticle. After the TWS1 precursor is activated by the endosperm-expressed ALE1, the final products can leak back through cuticle gaps.

A question remains as to why the endosperm-originated signals are needed for embryo development. One reason for this arrangement might lie in chromatin structure-based mechanisms. For example, it was shown that the timing of endosperm cellularization is epigenetically controlled [21]. Another study points out that the endosperm adopts a distinct high-order chromatin structure that differs from other cell types in other plant tissues [39]. It was speculated that genomic imprinting due to such unique arrangement in the endosperm chromatin structure allows the onset of gene activation of several important regulators prior to that of embryo [39]. However, understanding the detailed molecular mechanisms of the embryo-endosperm interaction requires more investigation. Studies on the distinct epigenetic environments in the endosperm and embryo would provide more insights into the tissue-specific regulatory networks. It was also plausible that such an arrangement allows a spatial separation of the embryo and its regulators and as such it would ensure that embryo development would not proceed until after a successful fertilization [15].

## 5. Endosperm and Embryo Dialogue, More to Discover

Endosperm development is not always destined to degeneration. In fact, the mechanisms described here in the model plant Arabidopsis may not represent those involved in other angiosperms. In monocots, especially in cereals, the endosperm persists after cellularization and undergoes PCD without degeneration. The distinct fates of endosperm development lead to differential volume relationships between endosperm and embryo in the two seed types [40]. In Arabidopsis, the main storage reserves are accumulated in the embryo, while in the cereal seeds it is the endosperm that accumulates storage products. Whether dialogues between endosperm development and embryogenesis in monocot and dicot species are different is an interesting question to be addressed.

Recent studies on *LEC1* in the two types of seeds might provide some clues. As described above, our research demonstrated that LEC1 in Arabidopsis expressed earlier in the endosperm, then transported to the embryo to facilitate embryo maturation [15]. In rice there are two homologues of *LEC1*, *OsNF-YB9* and *OsNF-YB7*. A recent study shows that heterologous expression of either *OsNF-YB9*, localized in the endosperm, or *OsNF-YB7*, expressed in the embryo, in Arabidopsis *lec1-1* plants complements the *lec1* defects [41]. In Arabidopsis, exclusive expression of *LEC1* in the endosperm of *lec1-1* would suffice for rescuing the *lec1-1* phenotypes. In rice, knocking out the embryo-expressed *OsNF-YB7* causes seed lethality even though the *OsNF-YB9* was still expressed in the endosperm, suggesting that the endosperm-expressed LEC1 cannot substitute the function of *OsNF-YB7*. Given that *OsNF-YB9* is capable of rescuing *lec1-1* seed defects in Arabidopsis, one of the possible explanations is that *OsNF-YB9* could not be mobilized from endosperm to embryo in rice, or in a manner that can sufficiently replace the embryo-expressed *OsNF-YB7*. 

In an evolutionary sense, the nuclear endosperm of cereals and Arabidopsis are not homologs, likely as a result of independent evolution from a maternal cellular megagametophyte tissue to a biparental endosperm [7]. Interestingly, a dicot species castor bean (*Ricinus communis*) produces albuminous seeds and exhibits a significant feature in endosperm development. Castor bean endosperm persists in the mature seeds which does not undergo PCD until upon seed germination [42]. Future investigation is warranted to address important questions on this front, e.g., why does the endosperm remain in the mature seeds of castor bean, and whether endosperm–embryo communication is necessary for endosperm to initiate PCD. Efforts are also needed to discover other key players involved in the communication.

Currently, the INTACT system makes it more accessible to isolate endosperm or embryo nuclei to profile their epigenome and transcriptome at an early stage [43,44,45]. Recent Single-cell RNA sequencing (scRNA-seq) allows us to understand seed developmental events at the single-cell or single-nucleus resolution [46,47]. Use of the INTACT system followed by chromatin immunoprecipitation sequencing or scRNA-seq will provides us an epigenomic and transcriptomic map to better understand endosperm–embryo communication.

## 6. Concluding Remarks

As summarized above, we have witnessed remarkable progresses in understanding the complex relationships and molecular communications between endosperm and embryo development in the model plant species. Endosperm supports embryogenesis not only by providing nutrients and growth regulators but also by regulating the embryo growth and maturation process through mobile protein signals. Creative experimental designs and advanced technologies will be necessary to gain more insight into when and how the endosperm and embryo communicate during seed development. As endosperm is a major nutrient resource, these findings would provide fundamental knowledge and inspirational strategies for seed engineering.
